# Diagnostic Efficacy of Four Methods for Locating the Second Mesiobuccal Canal in Maxillary Molars

**DOI:** 10.22037/iej.v13i2.16564

**Published:** 2018

**Authors:** Mariana De Carlo Bello, Camilla Tibúrcio-Machado, Clacir Dotto Londero, Fernando Branco Barletta, Carlos Heitor Cunha Moreira, Cláudia Medianeira Londero Pagliarin

**Affiliations:** a *Lutheran University of Brazil, Cachoeira do Sul, RS, Brazil****; ***; b * Universidade Federal de Santa Maria (UFSM), Santa Maria, RS, Brazil*;; c *Universidade Luterana do Brasil (ULBRA), Canoas, RS, Brazil*

**Keywords:** Cone-beam Computed Tomography, Diagnosis, Data Accuracy, Endodontics, Root Canal Therapy

## Abstract

**Introduction:**

The major cause for failure of root canal therapy is the inability to recognize the presence of all canals of the root canal system. Auxiliary tools, such as magnifying loupe, operative microscope and computed tomography (CT) images are used to facilitate the location of canals. The objective of the present survey was to evaluate the diagnostic efficacy of four methods for detecting the second canal of mesiobuccal roots (MB2) of permanent maxillary molars.

**Methods and Material:**

A total of 147 extracted human maxillary molars were assessed. The floor of the pulp chamber was inspected by an endodontist to find MB2 canals. Analyses were performed without magnification (direct visual method), using a loupe (with 3.5× magnification), and using a microscope (with 16× magnification). A fourth analysis was conducted using cone-beam computed tomography (CBCT) images. Teeth were sectioned horizontally into three parts (cervical, medial and apical thirds) to confirm the presence of MB2 canals (reference standard method). Sensitivity, specificity, and positive/negative predictive values were calculated for each method.

**Results:**

No statistically significant differences were observed in the frequency of MB2 found between the microscope and the reference standard or between CBCT and the reference standard. CBCT had higher sensitivity (0.88), specificity (0.88), positive (0.84) and negative (0.91) predictive value than the other three methods.

**Conclusion:**

CBCT was the most accurate method for detecting the MB2 and it had a diagnostic efficacy similar to that of the reference standard method.

## Introduction

The success of endodontic treatment depends on the correct identification of all canals present in the root canal system, adequate chemical-mechanical cleaning and hermetic seal obturation [1]. However, precise location of all canals remains a challenge that demands skill and expertise from the dental practitioner [2]. 

Root canal anatomy of maxillary molars is extremely complex and the mesiobuccal root exhibits a second canal in 25%-96% of the first molars [3, 4] and 11.53%-93.7% of the second molars [4, 5]. The inability of practitioners to locate these canals could partially explain the high levels of failure of endodontic treatment of teeth that have the second mesiobuccal (MB2) canal [6-8]. Also, the use of extra devices can explain the higher detection rate of additional canals [9].

The method traditionally used to locate root canals is direct visual inspection [10]. However, the efficacy of this method is closely related to the examiner’s skill and knowledge of root canal anatomy [11, 12]. In an attempt to facilitate location of accessory canals and reduce treatment failure rates, technological resources such as loupes, microscopes and cone-beam computed tomography (CBCT) have been introduced into endodontic practice. Loupes and microscopes are magnifying devices that can be applied in the dental office, allowing better visualization of canal entrances [13, 14]. Besides, use of microscope improves the illumination of the pulp floor, contributing for identification of extra canals [14]. However, the high cost of these technologies and hours of practicing to be able to operate them can be pointed as some disadvantages [15]. CBCT provides 3D-images from the root anatomy and it is a helpful tool for better visualization over the complexity of the root canal system [16]. A limitation of the method is that patients are subjected to a certain radiation dose. The American Association of Endodontists (AAE) and American Association of Oral Maxillofacial Radiology (AAOMR) recommend that the CBCT should not be used in the endodontic routine. However, if necessary, it is preferable to obtain acquisitions with small FOV and voxel aiming to reduce the radiation dose [17].

Even though many studies have demonstrated the ability of different methods to locate the presence of MB2 [9, 10, 12, 17-27], none has evaluated the diagnostic efficacy of these methods. Therefore, it is important to determine the sensitivity, specificity, and positive and negative predictive values of different methods for detection of MB2. The aim of this study was to evaluate the diagnostic efficacy of four methods commonly used in dental practice (direct visual inspection, use of loupe, use of microscope, and CBCT) to detect MB2 canals in permanent maxillary molars in comparison with the reference standard method of root cross-sectioning.

## Materials and Methods

A total of 147 extracted human permanent maxillary first and second molars obtained from the permanent tooth bank at Universidade Federal de Santa Maria (UFSM, Santa Maria, Rio Grande do Sul, Brazil) were analyzed *in vitro*. Information about donor’s age, sex, and skin color was not available. The study was approved by the UFSM Research Ethics Committee (protocol no. 97537). 

The teeth were cleaned using a Gracey curette 1/2 (Neumar, São Paulo, Brazil) to remove tissues at the root surface. Subsequently, the teeth were sterilized in an autoclave at 121^º^C and 1 ATM for 30 min, and then stored in purified filtered water (Asfer, São Paulo, Brazil) at 4^º^C until subsequent procedures. 

All access cavities were prepared using a size 1012 diamond bur (KG Sorensen, São Paulo, Brazil) and Endo Z bur (KG Sorensen, São Paulo, Brazil) mounted on a high-speed handpiece, with cooling. The floor of the pulp chamber, where the MB2 was expected to be located, was refined using ultrasonic tips (T3-S, Schuster, Santa Maria, Brazil) with light apical pressure to remove any calcifications present at the canal entrances. Following cavity preparation, the teeth were placed in trays (Angelus, Londrina, Brazil), which were filled with condensation silicone (Clonage, DFL, Rio de Janeiro, Brazil) up to the cemento enamel junction.

CBCT images were obtained using a Pax-Uni 3D unit (Vatech, Gyeonggi-do, South Korea) operating at 85 kVp, 4.0 mA, 8 sec. Voxel size was 0.125 mm and the field of view was 8×5 cm. All images were independently analyzed in a dark room by two endodontists trained (M.C.B and C.S.T) in advance. Cases of disagreement were reanalyzed collectively until consensus was reached. Images were analyzed using E2 3D software, version 1.2.5.3. Image contrast and brightness levels were adjusted using a tool available on the program to ensure adequate/consistent image quality. The number of canals present in the mesial root of each maxillary molar was determined based on CBCT axial scans.

The other three analyses (direct visual inspection, loupe and microscope) were carried out following a random order for each tooth by a calibrated endodontist who was blinded for previous diagnostic results. The three methods were employed as follows: 1) with no magnification tools (direct visual method); 2) using a loupe with 3.5× magnification (Bio Art, São Paulo, Brazil); and 3) using an optical microscope under 16× magnification (Aliance, São Paulo, Brazil). With each method, the floor of each pulp chamber was inspected to identify MB2 canals using endodontic Rhein probes (Golgran, São Paulo, Brazil). The presence of the MB2 canal was confirmed using a #10 K file (Dentsply Maillefer, Ballaigues, Switzerland) that penetrated the cervical third canal.

Finally, the mesiobuccal roots were cross-sectioned into three thirds (cervical, middle and apical) using diamond discs at low speed. These sections were analyzed by an endodontist under a stereomicroscope at 10× magnification, to confirm the presence of MB2 (reference standard method). MB2 was defined as present if the canal was visualized in the three sections.

Intra-observer reproducibility was checked by repeating all four analyses in a 20% subset of the sample (29 teeth). Intra-observer agreement for the four methods (direct visual inspection, loupe, microscope and CBCT) and for the reference standard, and inter-observer agreement for the CBCT method were calculated using Cohen’s kappa. Sensitivity, specificity, and positive and negative predictive values were calculated for each method by comparison with the reference standard (root cross-sectioning). Diagnostic efficacy results were expressed as percentages with 95% confidence interval (95% CI). Differences in the frequency of MB2 between groups were tested using McNemar test. The significance level was set at 5%. Statistical analyses were performed using the Statistical Package for the Social Sciences version 18.0 (SPSS, Chicago, IL, USA) and the Medcalc Software (Medcalc Software bvba, Ostend, Belgium).

## Results

Intra-observer agreement (Cohen’s kappa) was 1.0 for the direct visual, loupe, microscope and reference standard methods, 0.86 for CBCT examiner 1 and 0.92 for CBCT examiner 2. Inter-observer agreement for CBCT image analysis was 0.75. For the CBCT analysis, cases of disagreement were discussed collectively until consensus was reached.

MB2 was identified by cross-sectioning (reference standard) in 41.5% of the samples, which was significantly higher than the identification by direct visual (29.25%) and loupe (30.61%) methods (*P*<0.05). However, microscope (34.69%) and CBCT (43.54%) showed no difference in the MB2 detection comparing to the reference standard (Table 1).

The accuracy of CBCT in detecting MB2 canal was 88%, while microscope, loupe and direct visual showed accuracy of 62%, 62%, and 58%, respectively. Table 2 summarizes the results of sensitivity, specificity, positive and negative predictive values, and their respective confidence intervals for each of the four diagnostic methods investigated.

## Discussion

Many methods are available to aid clinicians in detecting MB2 canals, but no studies have evaluated the diagnostic efficacy of direct visual inspection, inspection using loupe, inspection using microscope, and analysis of CBCT images. The present study adds to the current body of knowledge by presenting sensitivity, specificity, positive and negative predictive values for these four methods in comparison with root cross-sectioning (reference standard method). 

It is worth mentioning that when a method is used to determine its ability in detecting what it is looking for, measuring its diagnostic accuracy is useful to judge the options and choose the best one [28]. The results obtained in this study showed that microscope method was not more accurate than direct visual inspection or inspection using loupe. Additionally, analysis of CBCT images proved to be a highly accurate method for detecting MB2 canals, with diagnostic accuracy similar to that of the reference standard method. It should be noted that accuracy is defined as the method ability to determine true positive and true negative results simultaneously.

In comparison with CBCT, the direct visual and loupe methods yielded unsatisfactory results for MB2 canal detection, as their sensitivity values were inferior to those found for CBCT. In the present analysis, sensitivity indicates the method’s ability to detect a MB2 when it is in fact present. Low sensitivity values are associated with a high number of false negative results, which could lead to failure of endodontic treatment of maxillary molars as a result of an undetected fourth canal. In contrast, specificity indicates the method’s ability to not detect the MB2 when it is not in fact present. The high specificity values found in the present study point to a low rate of false positive results for the methods investigated. Sensitivity and specificity values are important because they show whether a test is comparable to a reference standard.

From a clinical perspective, the diagnostic values with the greatest relevance are the positive and negative predictive values, because they provide information on a test power, *i.e.,* on the probability of the test result being true. Therefore, in the present study, positive predictive values indicate the probability that MB2 canals detected by the method are truly present (a low positive predictive value is associated with false positive results). This could have occurred in teeth in which the MB2 was identified, but the canal could not be negotiated. In turn, negative predictive value indicate the probability of true negative values not identified by the test. In this study, a high negative predictive value was found for CBCT, meaning that there is a high probability that the results of this test are correct, *i.e.,* that MB2 canals were actually absent in very few cases in which the CBCT test showed them to be present.

**Table 1:** Frequency of MB2 canals according to the four methods tested and the reference standard (root cross-sectioning) (*n*=147)

**Method**	**Absolute frequency (%)**
**Direct visual**	43 (29.25)*
**Loupe**	45 (30.61) *
**Microscope**	51 (34.69)
**CBCT**	64 (43.54)
**Cross-sectioning**	61 (41.50)

*MB2=second canal of mesiobuccal roots; CBCT = cone-beam computed tomography; *

* *Statistically significant differences between the method and reference standard*

**Table 2:** Sensitivity, specificity, positive and negative predictive values (expressed as percentages) and 95% confidence intervals for each diagnostic method compared to the reference standard (root cross-sectioning)

**Method**	**Sensitivity**	**Specificity**	**PPV**	**NPV**
**Direct visual**	34.43 (22.73-47.69)	74.42 (63.87-83.22)	48.84 (33.31-64.54)	61.54 (51.49-70.91)
**Loupe **	40.98 (28.55-54.32)	76.74 (66.39-85.18)	55.56 (40.00-70.36)	64.71 (54.62-73.91)
**Microscope **	45.90 (33.06-59.15)	73.26 (62.62-82.23)	54.90 (40.34-68.87)	65.62 (55.23-75.02)
**CBCT **	88.52 (77.78-95.26)	88.37 (79.65-94.28)	84.37 (73.14-92.24)	91.57 (83.39-96.54)

*PPV=positive predictive value; NPV=negative predictive method; CBCT=cone-beam computed tomography*

The present results showed the diagnostic accuracy of the microscope was similar to the visual and loupe methods. This similarity may be because of the expertise of the examiner responsible for the procedures. According to Corcoran, *et al*. [12] the examiner’s ability to locate root canals is largely dependent on clinical experience. Other possible explanation is that the removal of dentin excess from the canal entrances, as recommended by Kulild and Peters [29], also contributed to the similarity between the visual, loupe and microscope methods. Other findings have also suggested that difficulties in locating MB2 canals are primarily associated with the presence of calcifications [25, 30, 31]. On the other hand, the light apical pressure applied by the ultrasonic tip may not had removed calcifications above the MB2 in all specimens. Zhang *et al*. [32] in a sample of 1008 maxillary first molars found that 24% of the MB2 identified by CBCT were not at the cementoenamel junction, justifying why the results of the present study showed worse diagnostic accuracy for the microscope comparing to CBCT.

When comparing the frequency of MB2 found by the microscope with standard reference, no significant difference was observed. However, comparing visual method versus standard reference and loupe versus standard reference, the MB2 prevalence was significantly different. This is because the microscope showed more true and false positive values than the other two methods, which increased the frequency of MB2 by using the microscope. 

The high diagnostic values obtained for CBCT in this study attest to the use of this method as a new reference standard, especially in clinical studies, in which root sectioning is impossible. Accordingly, a previous study stated that CBCT is an absolutely reliable method for the detection of MB2 canals when compared with cross-sectioning of the roots [33]. CBCT allows clear visualization of the morphology of the mesial root of the maxillary molars, from the cervical to the apical third [33]. In our study, the good kappa value obtained for inter-observer agreement (0.75) reduced measurement bias and suggests that both examiners were consistently trained for the interpretation of images. Moreover, in cases where both disagreed, the presence/absence of the MB2 was discussed until reach a consensus. 

CBCT is a noninvasive radiographic method that produces three-dimensional, high-resolution images [19]. Nevertheless, the method has certain limitations. For example, the presence of artifacts created by metallic restorations may compromise image quality. This could explain why CBCT identified a larger number of MB2 than the reference standard method. Additionally, the radiation dose the patient is subjected limits its use in the endodontic routine and should be recommended with precaution [17].

*In vitro* studies have some limitations and translating their results to the clinical situation seems to be inappropriate. However, the recent literature supports our findings and points out that CBCT is reliable tool for detecting missing canals *in vivo* [34-36]. It is extremely important that dental professionals dedicate effort, knowledge and time to locating and preparing MB2 canals. Failure to detect this canal could lead to pathological complications and a subsequent need for endodontic retreatment [37]. According to the present results, the direct visual method, the use of loupe and microscope had low sensitivity, which possibly explains the high rate of endodontic failure caused by unidentified root canals [2, 7, 27, 38]. Even though the microscope have not shown high diagnostic values for detecting MB2, it is a useful device, because it facilitates the identification of root fractures and cracks [39], aids in the removal of posts [40] and during endodontic surgery [41]. CBCT, in turn, could be used as a supplementary tool in cases in which MB2 is not found using the methods available in the dental office.

## Conclusion

The present findings showed that the use of loupe and microscope are not more accurate methods when compared to direct visual inspection for detecting MB2 canals. Conversely, CBCT proved to be an accurate and reliable method for detecting MB2 canals.
